# External and Internal Shame in people with migraines

**DOI:** 10.1192/j.eurpsy.2023.1300

**Published:** 2023-07-19

**Authors:** I. Georgiadis, K. Fountas, F. Malli, E. Dragioti, M. Gouva

**Affiliations:** 1 1st Neurosurgical Department, Iaso Thessalias Private Hospital; 2 Department of Neurosurgery, Faculty of Medicine, University of Thessaly; 3 Respiratory Disorders Laboratory, Faculty of Nursing, University of Thessaly, Larissa; 4 Laboratory of Psychology of Patients, Families & Health Professionals, University of Ioannina – School of Health Sciences, Ioannina, Greece

## Abstract

**Introduction:**

Migraine often leads to reduction of social power and prestige of the patients, hence leading further emotions of shame.

**Objectives:**

Exploring the role of external and internal shame in people with migraines.

**Methods:**

The sample consisted of 180 people, more specifically 140 people from the general population and 40 people who have been diagnosed with migraine and receiving treatment for migraine, who completed the following questionnaires voluntarily and anonymously: a) Migraine Experience Questionnaire and Headache Impact Test-6 (HIT-6), b) the Other As Shamer scale (OAS) c) the Experience of Shame Scale (ESS), and socio-demographic and self-reported questionnaire.

**Results:**

Patients scored higher level external Shame (OAS) rates (31.28 ± 6.98) than people from the general population who scored lower external Shame (OAS) rates (16.89 ± 10.00) with a statistically significant difference between them (p = 0.000). Also, patients scored lower-level internal shame (ESS) rates (45.58 ± 6.91) than people from the general population who scored higher internal shame (ESS) rates (53.36 ± 15.62) with a statistically significant difference between them (p = 0.003).

**Conclusions:**

Patients with symptoms of migraine show statistically higher level of external shame and lower level of internal shame and further study is considered necessary.

**Disclosure of Interest:**

None Declared

